# Isometric handgrip contraction increases tibialis anterior intrinsic motoneuron excitability in a dose‐dependent manner

**DOI:** 10.1113/EP092961

**Published:** 2026-02-03

**Authors:** Lucas Ugliara, Lucas B. R. Orssatto, Amilton Vieira, Gabriel S. Trajano

**Affiliations:** ^1^ Faculty of Physical Education University of Brasilia (UnB) Brasilia Brazil; ^2^ Centre for Sensorimotor Performance, School of Human Movement and Nutrition Sciences The University of Queensland Brisbane Queensland Australia; ^3^ Faculty of Health, School of Exercise and Nutrition Sciences Queensland University of Technology (QUT) Brisbane Queensland Australia

**Keywords:** EMG, motoneuron, neuromodulation, PIC, remote contraction, serotonin

## Abstract

The contribution of persistent inward currents (PICs) to motoneuron firing in the lower limb typically increases after a remote handgrip contraction, believed to result from diffuse serotonergic input increases in spinal cord. We investigated whether handgrip contraction intensity, duration and/or impulse would affect PIC estimates in tibialis anterior motoneurons. Multi‐channel electromyograms were recorded from the tibialis anterior of 21 participants (18–40 years), during dorsiflexions at 20% of the individuals’ maximal torque, before and after four handgrip conditions: (i) 80% of their maximal handgrip strength sustained for 15 s (80%15s); (ii) 40% sustained for 15 s (40%15s); (iii) 40% sustained for 30 s (40%30s); and (iv) no handgrip (Control). The PIC contribution to self‐sustained motoneuron firing was estimated with delta frequency (Δ*F*) using paired motor unit analysis. The ‘brace height’, normalised as a percentage of a right triangle (% rTri), was used to estimate the PIC effects on the non‐linearity of firing patterns, representing the neuromodulatory drive (metabotropic regulation of motoneuron excitability) onto the motoneurons. Δ*F* increased by 0.33 pulses per second (pps; 95% CI: 0.16–0.49, *d *= 0.47) after 40%30s and by 0.24 pps (0.09–0.38, *d *= 0.34) after 80%15s, but remained unchanged after 40%15s and Control. Similarly, brace height increased by 2.24% rTri (0.18–4.30, *d *= 0.20) after 40%30s and by 2.45% rTri (0.64–4.25, *d *= 0.22) after 80%15s, remaining unchanged after 40%15s and Control. The increase in the PIC contribution to motoneuron firing induced by a remote handgrip contraction is impulse dependent rather than intensity or duration dependent. The parallel increases in Δ*F* and brace height suggest augmented neuromodulatory input onto the spinal cord.

## Abstract (highlights)

- **What is the central question of this study?**
  Does the intensity, duration and/or impulse (force–time integral) of a handgrip contraction determine the change in motoneuron firing behaviour of muscle groups that are not directly involved in the task?
- **What is the main finding and its importance?**
  A certain contraction impulse level during an isometric handgrip task is necessary to increase persistent inward current estimates in tibialis anterior motoneurons, independent of the remote task's intensity (force) or duration (time). These findings improve our understanding of the influence of neuromodulatory–inhibitory inputs, which that affect motoneuron excitability, as key factors in voluntary muscle contractions.

## 1 INTRODUCTION

Motoneurons rely on a complex control system for excitability, with neuromodulation as a key component. Unlike ionotropic systems, which directly convert synaptic inputs into action potentials via ion channel opening, neuromodulation operates through neurotransmitters that bind to specific receptors, initiating intracellular signalling pathways that regulate motoneuron responsiveness (Heckman et al., 2009). This neuromodulatory influence facilitates the generation of strong persistent inward currents (PICs) in spinal motoneurons, which increase cell excitability, accelerating, amplifying and prolonging their discharge for a given excitatory input (Heckman, Johnson et al., 2008). This input–output gain mechanism can be adjusted based on serotonin (5HT) and noradrenaline (NE) released from the raphe nuclei and locus coeruleus, respectively, which project to the spinal cord (Heckman, Hyngstrom et al., 2008) and activate receptors on motoneurons’ dendrites. This activation promotes an intracellular response via second messengers, leading to the opening of L‐type Ca^2+^ and Na^+^ channels and facilitating PICs in a dose‐dependent manner (Johnson & Heckman, 2014). It has been suggested that the level of neuromodulatory input onto the motoneurons could be adjusted according to physical task demand (Heckman et al., 2009). Lower levels of monoaminergic drive would lack the facilitation in recruited motoneurons, minimising involuntary synaptic noise and allowing precise movements (e.g., threading a needle). Alternatively, moderate‐to‐maximal motor tasks (e.g., lifting heavy weights) demand higher monoaminergic drive and an increased state of PIC facilitation (Johnson & Heckman, 2014). This theory is supported by the observation that the firing rate of neurons in the caudal raphe nuclei is influenced by motor output intensity, such that 5HT release is proportional to locomotion demands (Fenstermacher et al., 2025; Veasey et al., 1995). Importantly, most current knowledge of neuromodulation effects on PICs and 5HT's role in motoneuron gain control comes from invasive animal experiments and computational simulations (Heckman et al., 2009; Hounsgaard et al., 1988; Lee & Heckman, 2000). However, translating these findings to humans remains challenging, requiring non‐invasive techniques.

In humans, researchers have used different strategies to investigate the contribution of 5HT and NE to PIC neuromodulation through indirect measures. Pharmacological trials involving drugs that alter the concentration of 5HT and NE in the synaptic cleft have demonstrated their influence on PIC estimates and indicators of motoneuron excitability (e.g., discharge rate, recruitment threshold) (D'Amico et al., 2013; Goodlich et al., 2023, 2024; Udina et al., 2010). Additionally, some studies have used muscle contractions of different intensities to theoretically increase monoaminergic input onto the spinal cord to understand its effects on PICs. Although some studies observed no increase (Afsharipour et al., 2020; Kim et al., 2020), others report that the PIC contribution to motoneuron firing increases with contraction intensity within the same muscle group (Mackay et al., 2023; Orssatto et al., 2021), while the effect appears greater at higher force levels (Škarabot et al., 2025). The experimental strategy of using remote contractions to modulate motoneuron excitability is grounded in the pioneering work of Wei et al. (2014), who showed that remote leg contractions increased force variance during a precision task with the palm or index finger. This effect was interpreted as 5HT‐mediated, as force variation decreased following the administration of cyproheptadine, a 5HT receptor antagonist, and increased after escitalopram, a selective 5HT reuptake inhibitor, suggesting 5HT may amplify both task‐related and noisy synaptic inputs, reducing motor precision. Building on this evidence, other studies have since employed remote handgrip contractions to theoretically increase 5HT availability and thereby amplify PIC estimates in lower‐limb muscles (Mackay Phillips et al., 2023; Orssatto et al., 2022). These findings align with evidence that the firing rate of neurons in the caudal raphe nuclei and locus coeruleus is influenced, respectively, by motor outflow intensity and arousal, such that 5HT and NE release is proportional to task or locomotion demands (Fenstermacher et al., 2025; Heckman, Johnson et al., 2008; Veasey et al., 1995). Collectively, these findings align with animal evidence and support the feasibility of indirectly investigating serotonin's contribution to motoneuron firing modulation through remote muscle contractions.

After establishing the efficacy of remote handgrip tasks in increasing 5HT input onto the spinal cord and facilitating tibialis anterior and soleus PICs (Mackay Phillips et al., 2023; Orssatto et al., 2022), the next step was to understand whether PIC responses are differently influenced by mechanical aspects of remote contractions (i.e., force intensity, duration and impulse). This rationale is supported by evidence that higher contraction intensities increase estimates of PIC contribution to self‐sustained motoneuron firing (e.g., delta frequency, Δ*F*) in the soleus, gastrocnemius (Orssatto et al., 2021) and tibialis anterior (Mackay et al., 2023), suggesting that intensity modulates PIC activation. Additionally, serotonergic neuron activity in the raphe nuclei of cats rises and is maintained during prolonged locomotion until the cat no longer maintains pace (Jacobs et al., 2002) implying that sustained motor activity may also enhance the PIC functional contribution (i.e., increase the estimated magnitude or effect of PICs on motoneuron excitability). To investigate this phenomenon, the present study used multi‐channel electromyography to assess motor unit (MU) discharge rates, and the paired‐MU technique to estimate the contribution of PICs to motoneuron firing (Gorassini et al., 1998; Mesquita et al., 2024) following handgrip contractions at different intensity levels and contraction durations. In addition, we estimated the non‐linearity of motoneuron discharge patterns caused by monoaminergic drive using a quasi‐geometric approach (‘brace height’) applied to the ascending phase of the MU firing rates (Beauchamp et al., 2023). Given the evidence that contraction intensity affects PIC estimates in motoneurons (Mackay et al., 2023; Orssatto et al., 2021) and higher motor output potentially elevates 5HT concentration in the spinal cord and potentially increases motoneuron excitability (Jacobs et al., 2002), we hypothesise that the combination of both greater remote contraction intensity and duration (i.e., impulse) will induce higher PIC estimates.

## 2 METHODS

### 2.1 Ethical approval

All procedures conformed to the ethical standards set by the latest revision of the *Declaration of Helsinki*, except for registration in a public database. All participants provided written informed consent prior to participation. The study was approved by the Queensland University of Technology Human Research Ethics Committee (Reference number: 6770).

### 2.2 Participants and ethical procedures

Twenty‐three young adults participated in this study (Table 1). Inclusion criteria required volunteers to be within the age range of 18–40 years, have no history of musculoskeletal injuries in the tested limbs, and not be using medications that could affect the monoaminergic system (e.g., beta‐blockers and selective serotonin reuptake inhibitors) (Thorstensen et al., 2024). Furthermore, participants were instructed to abstain from strenuous physical activities and alcohol consumption for 48 h before the testing session.

**TABLE 1:** Participant characteristics.

Characteristic	Value
Age (years)	31 (29, 33)
Sex (*n*)	
Male	15
Female	6
Body mass (kg)	79 (73, 86)
Height (cm)	177(173, 181)
BMI (kg/m^2^)	25 (24, 27)
Handgrip peak force (N)	437 (386, 488)
Dorsiflexion peak torque (N m)	54 (49, 60)

Participant characteristics data are presented as means (95% CI).

### 2.3 Study design and testing procedures

The dorsiflexion and handgrip contractions were performed using the ‘preferred leg for kicking a ball’ and the corresponding ipsilateral hand, respectively. For the dorsiflexion tasks, participants were positioned upright in an isokinetic dynamometer (Biodex System 4, Biodex Medical Systems, Shirley, NY, USA) with the knee fully extended (0°), seat reclined at 70° of hip flexion and ankle in anatomical position. For the handgrip tasks, a handgrip dynamometer (model MLT004/ST, ADInstruments, Bella Vista, Australia) was held with shoulder in the anatomical position at 0° flexion/extension, 0° abduction/adduction, and 10–15° external rotation, with the elbow flexed at 90°, while seated on the isokinetic dynamometer. A warm‐up was performed alternating between handgrip and ankle dorsiflexion tasks, and consisted of progressive contraction levels (20%, 40%, 60%, 80% of perceived maximal effort) for ∼5 s each. Two minutes after, they performed two 4‐s handgrip maximal voluntary contractions, with a 60‐s interval between attempts. They were given a 2‐min rest and then also performed two 4‐s dorsiflexor maximal voluntary contractions, with a 60‐s interval between attempts. Peak handgrip force and dorsiflexion torque were considered as the maximum value achieved during the maximal voluntary contractions. Thereafter, participants were familiarised with triangular‐shaped ramped contractions to 20% of their dorsiflexion peak torque at 2%/s rate of torque increase and decrease (10 s up and 10 s down). Visual feedback was standardised for all participants, provided through a 23‐inch computer monitor, *y*‐axis range from 0 to 22% of peak torque, position standardised at ∼150 cm from the participant, and the software window maximised. Participants were guided to closely follow the real‐time torque trajectory by keeping a small yellow ball close to the requested torque trajectory. They received the following explicit verbal instructions: ‘Gradually increase and decrease your force, using your whole foot – not just your toes – to keep the yellow ball on the triangular line, avoiding abrupt increases or decreases of force generation’. This relative torque level was chosen based on previous studies investigating the effects of a remote handgrip contraction on lower limbs Δ*F* (Mackay Phillips et al., 2023; Orssatto et al., 2022).

**FIGURE 1:**
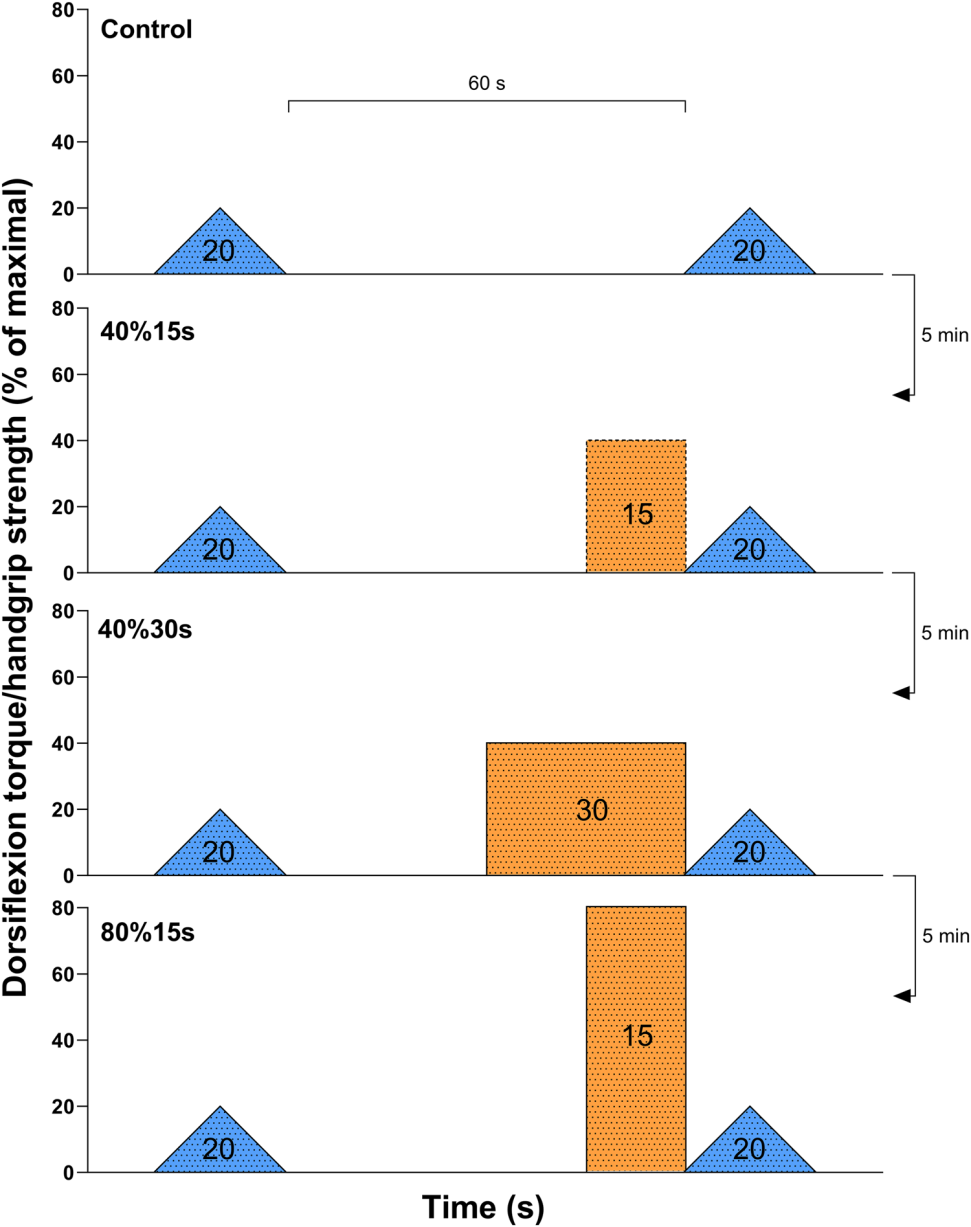
**Study protocol**. Blue triangles represent the triangular ramp‐shaped dorsiflexion contraction with the time between these two tasks set at 60‐s for all conditions. The dashed orange rectangle represents a handgrip contraction with half the contraction impulse of the solid orange rectangles. Conditions were performed randomly with 5 min rest interval.

Five minutes after familiarisation, four clusters of two sets of triangular‐shaped contractions, interspersed with either a resting control condition or three distinct handgrip contractions, were performed in a randomised order. During the control condition, the participants were asked to remain quiet and relaxed, avoiding moving or contracting any muscle for 60‐s in between the two triangular‐shaped contractions. For the three conditions involving a remote handgrip contraction, participants were requested to quickly reach a relative grip force level and sustain it for a given time; the three conditions were (i) handgrip contraction at 40% of their peak force sustained for 15 s (40%15s); (ii) handgrip contraction at 40% of their peak force sustained for 30 s (40%30s), and (iii) handgrip contraction at 80% of their peak force sustained for 15 s (80%15s). Conditions 80%15s and 40%30s were impulse matched, 80%15s and 40%15s were time‐matched, and 40%30s and 40%15s were intensity matched. This approach was adopted to allow us to investigate whether the increases in Δ*F* are affected by remote contraction time (40% sustained for 15 s vs. 30 s), intensity (40% vs. 80% sustained for 15 s), or impulse (40% sustained for 30 s and 80% sustained for 15 s vs. 40% sustained for 15 s). The time between the two triangular‐shaped contractions, performed before and after the control or handgrip conditions, was standardised at 60‐s. Thus, a 30‐s waiting period preceded the handgrip contraction lasting 30 s, while a 45‐s waiting period preceded the handgrip contraction lasting 15 s. The second triangular‐shaped contractions were performed immediately after the handgrip conditions. A 5‐min rest interval was adopted between conditions. Figure 1 illustrates the study protocol design. In case abrupt/steep increases or decreases (>5% of peak torque) of torque were observed during the ascending or descending phase of the triangular‐shaped contractions, the whole trial for the specific condition was excluded and repeated after 5 min. The participants were given only one extra attempt for each condition, when necessary. No further attempts were given to avoid fatigue, which can affect the outcomes obtained from the triangular contractions (Mackay et al., 2023). If the participant was not able to maintain consistency in the before and after triangular ramp‐shaped contractions (i.e., avoid abrupt/steep increases or decreases in torque) for both attempts in a given condition, they were excluded from the analysis for that condition. Finally, the impulse (area under the force curve) performed at the handgrip conditions was calculated using the trapezoidal rule in LabChart software (version 7.3, ADInstruments).

### 2.4 Multi‐channel electromyography recordings and analyses

The tibialis anterior skin area was prepared by shaving and cleansing with 70% isopropyl alcohol. A semi‐disposable 64‐channel electrode grid with an interelectrode distance of 8 mm (GR08MM1305, OT Bioelettronica, Turin, Italy), attached to a bi‐adhesive foam layer and coated with a conductive paste (Ten20, Weaver and Company, Aurora, CO, USA), was positioned over the most prominent part of the tibialis anterior. The grounding electrode comprised a dampened strap (WS2, OT Bioelettronica) placed around the ankle joint. Multi‐channel electromyograms were recorded during the triangular ramp‐shaped contractions using monopolar mode, amplified (256×), subjected to band‐pass filtering (10–500 Hz), and digitally sampled at 2048 Hz, with a 16‐bit wireless amplifier (Sessantaquattro, OT Bioelettronica), interfacing with OTBioLab+ software (version 1.3.0., OT Bioelettronica) and were stored for subsequent offline analysis. The recorded data were processed offline using DEMUSE software (Holobar & Zazula, 2007). The signals were band‐pass filtered (20–500 Hz) through a second‐order, zero‐lag Butterworth filter. Subsequently, MU decomposition was performed using the convolutive kernel compensation (CKC) method (Holobar & Zazula, 2007; Holobar et al., 2014). Following decomposition, the same MUs were tracked across the before and after handgrip or control conditions, but not across conditions. The files for each contraction were concatenated and separation vectors (i.e., MU filters) were used to identify and generate the MU spike trains across the two contractions (Del Vecchio et al., 2019; Holobar & Zazula, 2007; Holobar et al., 2014). Then, a trained investigator (L.U.) examined the MU spike trains, and, when necessary, edited the discharge patterns of the MUs (Del Vecchio et al., 2020). Only MUs with a pulse‐to‐noise ratio ≥30 dB were retained for presenting higher reliability (sensitivity >90% and false alarm rates <2%) (Holobar et al., 2014). The edited MUs were quality‐checked by a researcher, blinded for each condition (L.B.R.O.) with extensive experience in decomposing, tracking and editing of MUs. When no MU was identified in both contractions for a given condition, the participant was not included in the respective condition analysis.

#### 2.4.1 Estimation of PIC contribution to motoneuron self‐sustained firing (ΔF) and peak discharge rate

**FIGURE 2:**
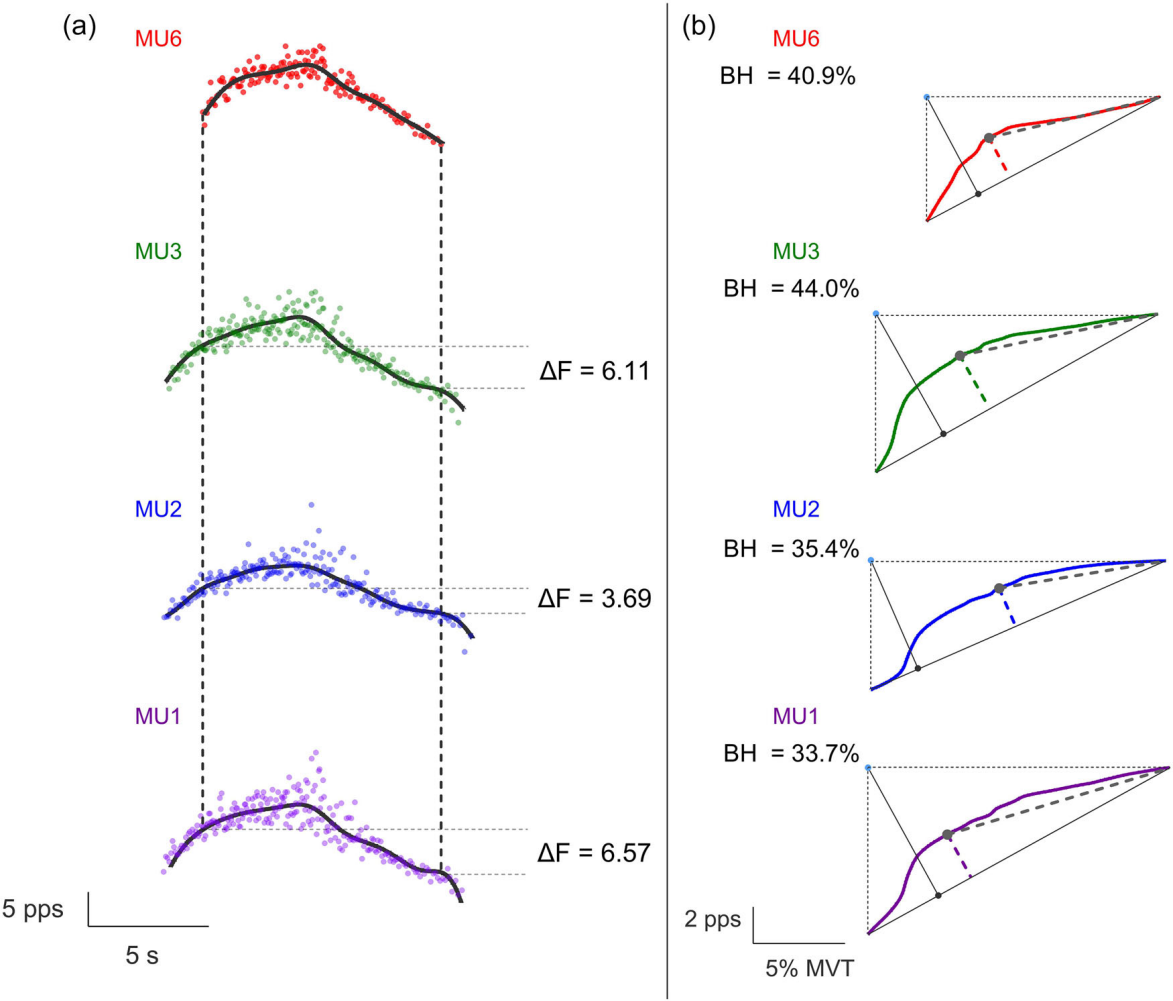
**Example of ΔF, brace height, and attenuation slope calculations**. (a) Red represents firing of the test unit (top), paired with the other three control units (green, blue and purple). Continuous black lines, SVR smoothed curve of the MUs; dashed vertical black lines, moment of recruitment and decruitment of the test unit; dashed horizontal grey lines, firing of control units at the moment of recruitment and derecruitment of the test unit, used to calculate Δ*F*. (b) Continuous coloured lines, SVR smoothed curve of the MUs; dashed coloured lines, brace height; continuous black line, orthogonal distance originating from the hypotenuse (blue dot) to the right triangle (black dot) formed by the moment of recruitment to derecruitment of each MU (dashed black lines); dashed grey lines, attenuation slope; grey dots, point where brace height inserts and attenuation slope starts.

The torque signal was filtered using a fifth‐order, low‐pass Butterworth filter with a cut‐off frequency of 10 Hz. Then, the MU discharge events were converted into instantaneous discharge rates and smoothed with a support vector regression machine learning fit (Beauchamp et al., 2022). PIC contribution to motoneuron firing was estimated using a paired MU analysis (Afsharipour et al., 2020). MUs with lower recruitment thresholds (control units) were paired with units of higher recruitment threshold (test units). Δ*F* was calculated as the change in discharge rates of the control MU from the onset of recruitment to the point of de‐recruitment of the test unit (Afsharipour et al., 2020) (Figure 2a). MUs were paired when the following criteria were obtained between control and test units: (i) rate‐to‐rate correlation threshold of *r* ≥ 0.7, (ii) test–control unit recruitment time >1 s, (iii) control unit's discharge rate at test unit recruitment minus the control unit's peak discharge rate >0.5 pulses per second (pps), and (iv) control unit derecruitment time > test unit derecruitment time (Afsharipour et al., 2020; Hassan et al., 2020; Vandenberk & Kalmar, 2014). Δ*F* values calculated for each individual test unit were averaged across control units to yield a singular Δ*F* value for each corresponding test MU. In the case that no MU pair was identified in both contractions for a given condition, the participant was not included in the analysis for that respective condition. The highest value derived from the support vector regression fit curve was determined as peak discharge rate. The relative torque (%) produced during the recruitment time of each MU was identified as MU recruitment threshold.

#### 2.4.2 Estimation of neuromodulatory and inhibitory drive contributions to MU firing changes

We used the quasi‐geometric approach to estimate the neuromodulatory and inhibitory drive contributions to motoneuron firing, as proposed by Beauchamp et al. (2023) (Figure 2b). First, we generated a straight line (hypotenuse) from the discharge rate at MU recruitment to the peak discharge rate of the support vector regression (SVR) smoothed curve. Then, we calculated the ‘brace height’, which represents the maximum deviation of the smoothed discharge rates trace from linearity (hypotenuse). The maximal orthogonal distance between the hypotenuse and the smoothed MU discharge trace was considered the brace height. Thereafter, brace height was normalised as a percentage of the maximal orthogonal distance between the straight line and a right triangle in which sides originate from the hypotenuse. We also calculated the ‘attenuation slope’, which is proposed to be associated with the inhibitory input effect on MU discharge (Beauchamp et al., 2023). Attenuation slope was calculated from the brace height insertion on the smoothed discharge rates to the peak discharge rates of the ascending phase.

### 2.5 Data and statistical analyses

Linear‐mixed effects models were investigated utilising the *robustlmm* package (Kuznetsova et al., 2017). We estimated marginal mean differences between handgrip conditions in Δ*F*, peak discharge rate, brace height and attenuation slope along with 90% and 95% confidence intervals (CI), using the *emmeans* package (Lenth et al., 2023). Each MU was treated as repeated measure and nested for each participant, including a random intercept for each participant to consider for the correlation between repeated observations on each individual (1 participant/MU ID). For Δ*F*, four distinct models were fitted: (i) time and condition were included as fixed effects and a random intercept and slope (Δ*F*) for each participant; (ii) similar to model 1, but included peak discharge rate as covariate; (iii) similar to model 1, but included recruitment threshold as covariate; and (iv) similar to model 1 but included both peak discharge and recruitment threshold as covariates. All four models resulted in similar outcomes; thus we presented the findings derived from Model 4, which exhibited a superior fit (as indicated by the lowest Akaike's information criterion and Bayesian information criterion). We performed an additional analysis by adding sex as fixed effect to Δ*F* to investigate a possible effect of sex on results, but no time by condition by sex interaction effect [β = 0.32 (–0.29, 0.92) pps, SE = 0.32; *t* = 0.31] and main effect of sex [β = –0.80 (–1.76, 0.16) pps, SE = 0.49; *t* = −1.64] were observed, so sex was removed from the model. For all other variables except by recruitment thresholds (i.e., peak discharge rate, brace height and attenuation slope), a model with time and condition as fixed effects and recruitment thresholds as covariate was employed. For recruitment thresholds and derecruitment thresholds, the model included time and condition as fixed effects. We also used the *robustlmm* package to check the consistency of the participants in performing the triangular‐shaped contractions by comparing the area formed between the performed and the requested torque trace paths, and results are presented in Supplementary Material 1. In addition, the mean impulse for each condition was compared using the *lmerTest* package for linear mixed effects models analysis. Pairwise *post hoc* analysis was used for significant effects observed. Cohen's *d* effect sizes were computed for the differences between after and before handgrip conditions, leveraging the population standard deviation (σ) estimated from corresponding robust linear mixed‐effects models as the denominator (i.e, *d *= mean difference/σ). For interpretation, we considered Cohen's *d* < 0.20 trivial; 0.20–0.49 small; 0.50–0.79 moderate; ≥0.80 large. A significance threshold of 5% (α level) was adhered to for all tests. All the analyses were conducted in a free software environment (RStudio, version 2024.12.1). The complete dataset and corresponding R script are accessible at https://github.com/lugliara/PICs.

## 3 RESULTS

### 3.1 Participant characteristics and MU identification

One female participant was excluded from all the analyses due to inconsistencies in performing the ramp‐shaped triangular contractions in all the conditions and another female participant was excluded because no MUs were identified at any condition. The final analysis included data from 21 participants. Among these 21 participants, all of them were included in the control and 80% of peak force for 15 s conditions (80%15s). For the 40% of peak force for 15 s (40%15s), data from five participants were excluded from the analyses because they were unable to consistently follow the force trace path during the triangular‐shaped contractions without steep increases and decreases of force production (*n* = 3), or MUs did not meet essential prerequisites to form pairs (*n* = 2). For the 40% of peak force for 30 s (40%30s), data from three participants were excluded from the analyses because they were unable to follow the feedback path (i.e., detected abrupt/steep increases or decreases >5% of peak torque) during the triangular‐shaped contractions (*n* = 2) or no MUs were detected (*n* = 1). In order to investigate the influence of excluding some trials on our results, we performed a sensitivity analysis including only the participants who successfully concluded all four conditions (*n* = 14). The main findings remained largely consistent with the original analysis. Although a Time by Condition interaction effect for Brace Height was no longer statistically significant, this does not affect the interpretation of Δ*F*, which is our primary outcome. The results for this analysis are presented in Supplementary Material 2 and the data have been made publicly available on https://github.com/lugliara/PICs for transparency.

There were 341, 249, 245 and 316 MUs which were matched before and after the control [mean (95% CI) = 16.2 (13.9, 18.6), *n* = 21], 40%15s [15.6 (13.3, 17.8), *n* = 16], 40%30s [13.6 (10.8, 16.4), *n* = 18] and 80%15s [15.0 (12.2, 17.9), *n* = 21] conditions, respectively. This resulted in 199 [9.5 (7.8, 11.2), *n* = 21], 146 [9.1 (7.2, 11.1), *n* = 16], 137 [7.6 (5.7, 9.6), *n* = 18] and 191 [9.1 (6.8, 11.4), *n* = 21] test units, respectively.

#### 3.1.1 Handgrip contraction impulse

The impulse generated during the handgrip contractions during 40%30s [estimated marginal mean = 5639 (95% CI: 5054, 6224) N s] and 80%15s [5667 (95% CI: 5082, 6252) N s] were similar (*P* = 0.868), but superior to 40%15s [2933 (95% CI: 2348, 3519) N s, *P* < 0.001].

### 3.2 MU outcomes

#### 3.2.1 ΔF

**FIGURE 3:**
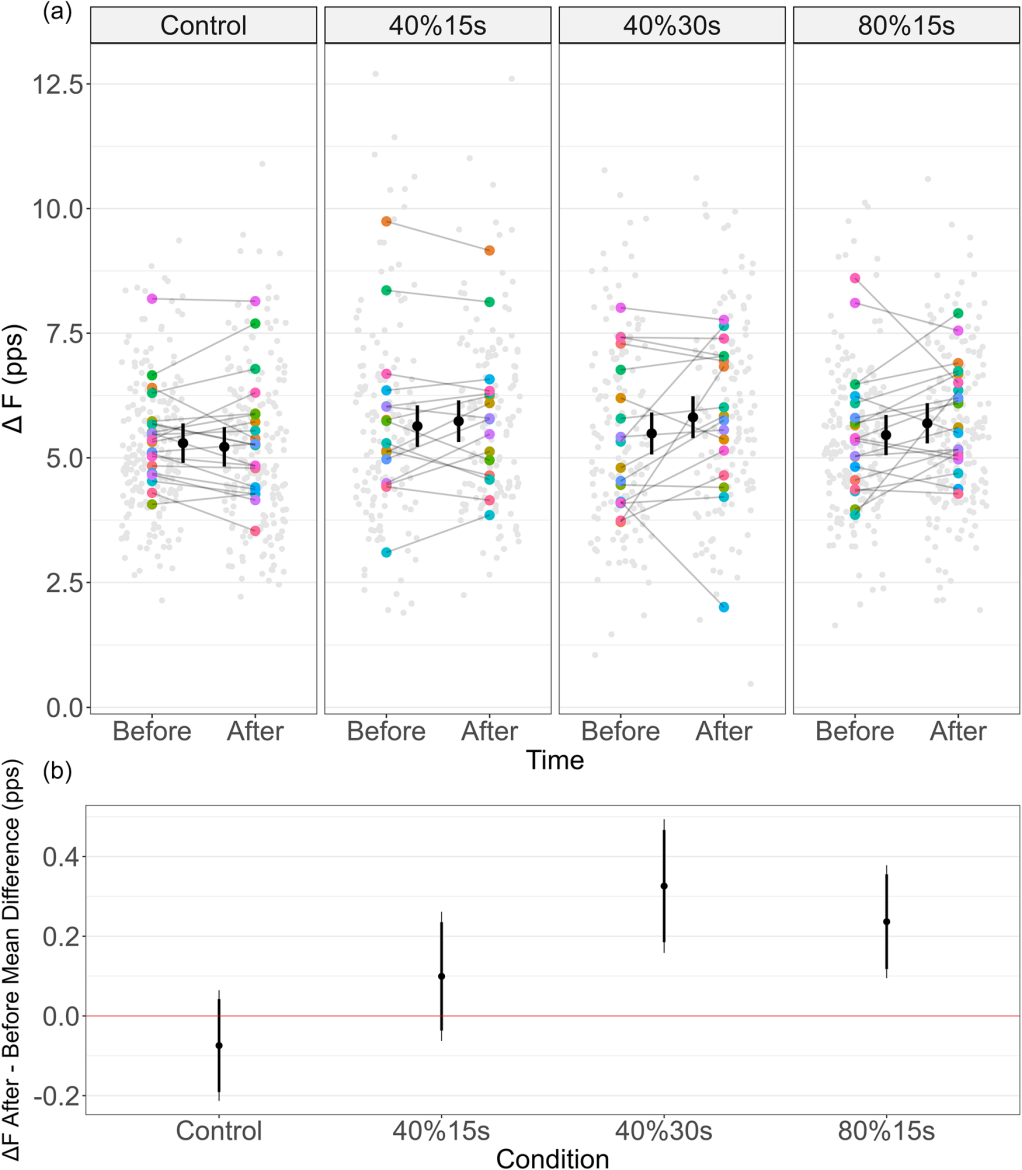
**Tibialis anterior ΔF in control and remote handgrip conditions**. (a) The estimated marginal means (black circles) and respective 95% confidence intervals are offset to the middle. Individual data points (averaged Δ*F* per participant) are coloured by participants and individual test units value are plotted in light grey. pps, pulses per second. Δ*F* remained unchanged before and after control and 40%15s conditions but increased at 40%30s and 80%15s. (b) Significant increases were observed on the handgrip at 40%30s and 80%15s conditions (not crossing the ‘zero’ red line). The thick inner line and the thin outer line represent the 90% and 95% confidence intervals, respectively.

A time by condition interaction effect was observed [β = −0.40 (−0.62, −0.18) pps, SE = 0.11; *t* = −3.60]. Δ*F* increased from before to after the intervention in 40%30s [0.33 (0.16, 0.49) pps, *d* = 0.47 (0.23, 0.72)] and 80%15s [0.24 (0.09, 0.38) pps, *d* = 0.34 (0.14, 0.55)], but remained unchanged in 40%15s [0.10 (−0.06, 0.26) pps, *d* = 0.15 (−0.09, 0.38)] and control [−0.07 (−0.21, 0.06) pps, *d* = −0.11 (−0.31, 0.09)] conditions (Figures 3a, b).

#### 3.2.2 Brace height

**FIGURE 4:**
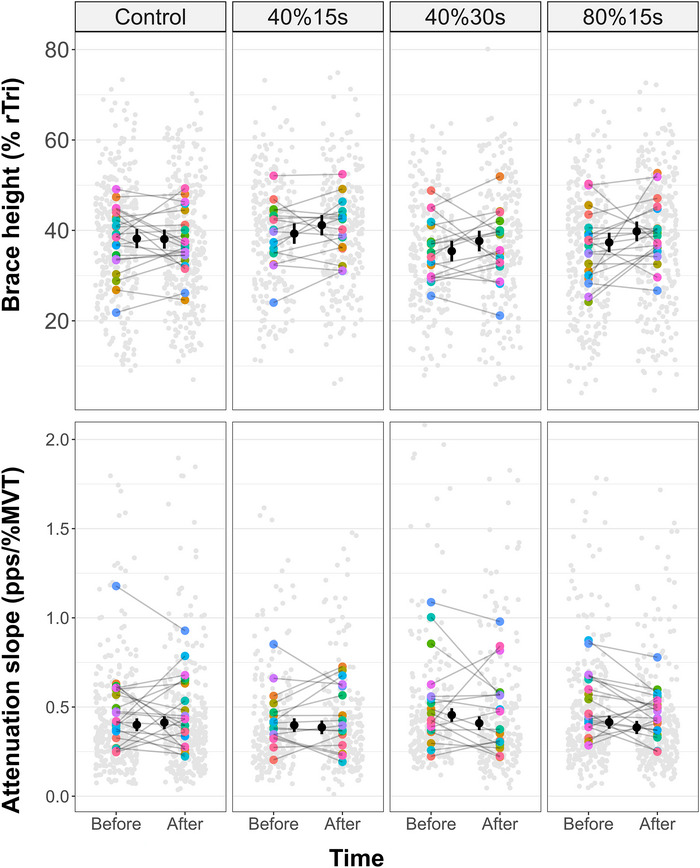
**Tibialis anterior brace height and attenuation slope in control and remote handgrip conditions**. The estimated marginal means (black circles) and respective 95% confidence intervals are offset to the middle. Individual data points (averaged brace height and attenuation slope per participant) are coloured by participants and individual motor units value are plotted in light grey. Maximal voluntary torque (MVT), maximum voluntary torque; % rTri, percentage of the right triangle; pps, pulses per second. Both brace height and attenuation slope remained unchanged before and after control and 40%15s conditions but, respectively, increased and decreased at 40%30s and 80%15s. Note, the *y*‐axis for attenuation slope has been limited to 2 pps/%MVT to enhance the visualization of data points and their 95% confidence intervals. A full‐range version of the figure is available at https://github.com/lugliara/PICs.

A time by condition interaction effect was observed [β = −2.56 (−5.06, −0.06)% rTri, SE = 1.27; *t* = −2.01]. Brace height increased from before to after the intervention in 40%30s [2.24 (0.18, 4.30)% rTri, *d* = 0.20 (0.02, 0.39)] and 80%15s [2.45 (0.64, 4.25)% rTri, *d* = 0.22 (0.06, 0.39)], but remained unchanged in 40%15s [1.86 (−0.15, 3.87)% rTri, *d* = 0.17 (−0.01, 0.35)] and control [−0.11 (−1.84, 1.61)% rTri, *d* = −0.01 (−0.17, 0.15)] conditions (Figure 4).

#### 3.2.3 Attenuation slope

A time by condition interaction effect was observed [β = 0.06 (0.02, 0.10) pps/% maximal voluntary torque (MVT), SE = 0.02; *t* = 2.73]. Attenuation slope decreased from before to after the intervention in 40%30s [−0.05 (−0.08, −0.01) pps/%MVT, *d* = −0.27 (−0.46, −0.08)] and 80%15s [−0.03 (−0.06, −0.00) pps/%MVT, *d* = −0.17 (−0.34, −0.01)], but remained unchanged in 40%15s [−0.01 (−0.04, 0.02) pps/%MVT, *d* = −0.07 (−0.26, 0.11)] and control [0.01 (−0.01, 0.04) pps/%MVT, *d* = 0.07 (−0.08, 0.23)] conditions (Figure 4).

#### 3.2.4 Peak discharge rates

A time by condition interaction was observed [β = −0.50 (−0.62, −0.37) pps, SE = 0.06; *t* = −7.64]. Peak discharge rates decreased from before to after the intervention only in control [−0.29 (−0.38, −0.21) pps, *d* = −0.52 (−0.67, −0.36)] and increased in the 80%15s [0.20 (0.11, 0.29) pps, *d* = 0.35 (0.19, 0.51)] condition, but remained unchanged in 40%15s [0.06 (−0.04, 0.16) pps, *d* = 0.10 (−0.08, 0.28)] and 40%30s [−0.09 (−0.19, 0.02) pps, *d* = −0.16 (−0.34, 0.03)] conditions.

#### 3.2.5 Recruitment thresholds

A time by condition interaction effect was observed [β = −0.89 (−1.17, −0.60)% of peak torque, SE = 0.15; *t* = −6.01]. Recruitment thresholds increased from before to after the intervention only in 40%15s [0.51 (0.29, 0.73)% of peak torque, *d* = 0.42 (0.24, 0.60)] and 40%30s [0.31 (0.09, 0.54)% of peak torque, *d* = 0.26 (0.07, 0.44)] conditions, and decreased in the control condition [−0.38 (−0.57, −0.19)% of peak torque, *d* = −0.31 (−0.47, −0.16)], but remained unchanged in the 80%15s condition [−0.06 (−0.26, 0.14)% of peak torque, *d* = −0.05 (−0.21, 0.11)].

#### 3.2.6 Derecruitment thresholds

A time by condition interaction effect was observed [β = 0.53 (0.26, 0.79)% of peak torque, SE = 0.14; *t* = 3.89]. Derecruitment thresholds decreased from before to after the intervention only in 40%15s [−0.68 (−0.88, −0.48)% of peak torque, *d *= −0.61 (−0.79, −0.43)] and 80%15s [−0.37 (−0.55, −0.19)% of peak torque, *d *= −0.33 (−0.49, −0.17)] conditions, but remained unchanged in control [−0.15 (−0.33, 0.02)% of peak torque, *d *= −0.14 (−0.29, 0.02)] and 40%30s [−0.15 (−0.36, 0.05)% of peak torque, *d *= −0.14 (−0.32, 0.04)] conditions.

Table 2 describes the before and after estimated marginal means and mean differences for each MU outcome and tested conditions.

**TABLE 2:** Estimated marginal means and mean differences (95% confidence interval lower and upper limits) for Δ*F*, peak discharge rates, recruitment thresholds, brace height, and attenuation slope for handgrip and control conditions.

	Δ*F* (pps)	Brace height (% rTri)	Attenuation slope (pps/%MVT)	Peak discharge rate (pps)	Recruitment threshold (% of peak torque)	Derecruitment threshold (% of peak torque)
Control						
Before	5.29 (4.90, 5.69)	38.2 (36.1, 40.3)	0.40 (0.36, 0.44)	17.2 (16.1, 18.2)	8.54 (7.16, 9.92)	7.60 (6.64, 8.46)
After	5.22 (4.82, 5.62)	38.1 (36.0, 40.2)	0.41 (0.38, 0.45)	16.9 (15.8, 17.9)	8.16 (6.79, 9.54)	7.45 (6.59, 8.31)
After − before	−0.07 (−0.21, 0.06)	−0.11 (−1.84, 1.61)	0.01 (−0.01, 0.04)	**−0.29 (−0.38, −0.21)**	**−0.38 (−0.57, −0.19)**	−0.15 (−0.33, 0.02)
40%15s						
Before	5.64 (5.22, 6.05)	39.3 (37.0, 41.6)	0.40 (0.36, 0.44)	17.1 (16.0, 18.1)	8.08 (6.66, 9.49)	8.10 (7.23, 8.98)
After	5.73 (5.32, 6.15)	41.2 (38.9, 43.4)	0.39 (0.35, 0.42)	17.1 (16.1, 18.2)	8.58 (7.17, 10.00)	7.42 (6.54, 8.30)
After − before	0.10 (−0.06, 0.26)	1.86 (−0.15, 3.87)	−0.01 (−0.04, 0.02)	0.06 (−0.04, 0.16)	**0.51 (0.29, 0.73)**	**−0.68 (−0.88, −0.48)**
40%30s						
Before	5.49 (5.07, 5.91)	35.4 (33.1, 37.7)	0.46 (0.42, 0.49)	17.4 (16.3, 18.4)	8.32 (6.91, 9.74)	7.49 (6.61, 8.36)
After	5.81 (5.39, 6.24)	37.7 (35.4, 39.9)	0.41 (0.37, 0.45)	17.3 (16.2, 18.3)	8.63 (7.22, 10.05)	7.33 (6.45, 8.21)
After − before	**0.33 (0.16, 0.49)**	**2.24 (0.18, 4.30)**	**−0.05 (−0.08, −0.01)**	−0.09 (−0.19, 0.02)	**0.31 (0.09, 0.54)**	−0.15 (−0.36, 0.05)
80%15s						
Before	5.46 (5.05, 5.86)	37.3 (35.2, 39.5)	0.412 (0.38, 0.45)	17.4 (16.4, 18.5)	9.00 (7.61, 10.40)	8.08 (7.21, 8.94)
After	5.69 (5.29, 6.10)	39.8 (37.6, 42.0)	0.39 (0.35, 0.42)	17.6 (16.6, 18.7)	8.94 (7.55, 10.33)	7.71 (6.84, 8.58)
After − before	**0.24 (0.09, 0.38)**	**2.45 (0.64, 4.25)**	**−0.03 (−0.06, −0.00)**	**0.20 (0.11, 0.29)**	−0.06 (−0.26, 0.14)	**−0.37 (−0.55, −0.19)**

Bold values indicate significant changes (95% CI not including zero). Δ*F*, Δ frequency; MVT, maximum voluntary torque; % rTri, percentage of the right triangle.

## 4 DISCUSSION

We investigated whether remote handgrip contraction characteristics – duration (40%15s vs. 40%30s), intensity (40%15s vs. 80%15s) and the impulse (40%30s and 80%15s vs. 40%15s) – affect tibialis anterior PICs. Quasi‐geometric analyses estimated neuromodulatory (i.e., brace height) and inhibitory (i.e., attenuation slope) contributions to PIC responses. The main findings were: (i) Δ*F* increased after the higher handgrip impulse conditions; (ii) brace height also increased under the same conditions; and (iii) attenuation slope consistently decreased. These findings suggest the mechanical impulse of a remote contraction was the primary driver of increased Δ*F*, likely via enhanced neuromodulation and shifted inhibitory pattern.

Changes in Δ*F*, brace height and attenuation slope between the two highest handgrip impulse conditions (80%15s and 40%30s) versus 40%15s and Control support our hypothesis, suggesting an impulse threshold might be necessary to induce tibialis anterior MU recruitment–derecruitment hysteresis. The small Δ*F* increases at the 40%30s (*d *= 0.47) and 80%15s (*d *= 0.34) conditions were similar to Mackay et al. (2023) in soleus (*d *= 0.30) and Orssatto et al. (2022) in tibialis anterior (*d *= 0.55), both using a similar 40%30s protocol. Such Δ*F* changes can be influenced by neuromodulatory and/or inhibitory inputs (Beauchamp et al., 2023). Monoamines are the main driver of PIC activity. In animals, voltage‐clamp experiments show PICs result from voltage‐gated L‐type Ca^2+^ (Eckert & Lux, 1976; Svirskis & Hounsgaard, 1997) and Na^+^ (Harvey et al., 2006; Schwindt & Crill, 1977) channel activation. 5HT and NE activating specific G‐protein coupled receptors at motoneurons’ dendrites and soma trigger intracellular signalling cascades, modulating these ion voltage‐gated channels (Harvey et al., 2006; Heckman et al., 2009; Perrier & Hounsgaard, 2003). In humans, oral amphetamine, which is assumed to enhance the presynaptic NE release, increased estimates of Δ*F* (Udina et al., 2010), suggesting similar neuromodulation to that observed in animals in humans. Wei et al. (2014) showed 5HT modulates motoneurons’ input–output gain via reuptake inhibitors and receptor blockers. Recently, multi‐channel electromyography (EMG) during voluntary ramped contractions demonstrated decreased MU excitability with 5HT blockers, measured via Δ*F* and/or other PIC estimates (Goodlich et al., 2023, 2024). Furthermore, higher Δ*F* has been observed at higher‐intensity ramped contractions (Mackay et al., 2023; Orssatto et al., 2021; Škarabot et al., 2025), suggesting greater voluntary muscle activity in humans is associated with higher PIC activity and, likely, higher 5HT and NE in the spinal cord. Therefore, the Δ*F* increase observed in this study after higher‐impulse handgrip contractions was likely driven by a transient monoamine rise; however, this mechanism was not directly tested and future studies with drugs affecting monoamine concentration might be able to address this mechanism.

We used brace height to estimate neuromodulatory contributions to motoneuron firing, as it was proposed to reflect increased serotonergic and/or noradrenergic input (Beauchamp et al., 2023). Thus, increases in brace height at 40%30s (*d* = 0.20) and 80%15s (*d* = 0.22) suggest remote handgrip contraction induced greater neuromodulation. In addition, PICs are highly sensitive to inhibition (Hultborn et al., 2003; Kuo et al., 2003), which controls unwanted movement that could arise from diffuse monoaminergic descending projections (Heckman et al., 2009). Spinal inhibition has been observed with small antagonist muscle length changes (Hultborn et al., 2003; Hyngstrom et al., 2007), tendon vibration (Matthews, 1966; Orssatto et al., 2022; Pearcey et al., 2022), passive muscle stretching (Trajano et al., 2014) and voluntary co‐contraction (Gomes et al., 2024), likely via disynaptic inhibitory circuits activated by muscle spindle Ia afferents (Crone et al., 1987; Kuffler et al., 1951). However, the consistency of ramp‐shaped contractions used in the present study before and after remote tasks makes Ia‐mediated changes unlikely. Inhibition input might also originate in supraspinal or segmental mechanisms, e.g., Renshaw cells (Hultborn & Pierrot‐Deseilligny, 1979), or nociceptive inputs (Heckman et al., 2009). We used attenuation slope to estimate inhibitory input influence since inhibition patterns might predict this measure (Beauchamp et al., 2023). The small and trivial decrease in attenuation slope at 40%30s (*d* = −0.27) and 80%15s (*d* = −0.17) might indicate a transition from a tonic inhibitory pattern to inhibitory commands that are more reciprocal to excitation (i.e., push–pull excitation–inhibition synaptic control) (Škarabot et al., 2025). However, caution is needed, as Δ*F* sensitivity to inhibition patterns depends on neuromodulation levels (Beauchamp et al., 2023). Additionally, MUs exhibiting larger brace height theoretically show lower attenuation slope (Mesquita et al., 2024). Thus, our findings suggest that, once a certain level of impulse is reached, remote contractions of either lower or higher intensity/duration similarly increase in the estimated PIC contribution to motoneuron self‐sustained firing (Δ*F*). This effect is likely driven by increased neuromodulation onto the spinal cord, as indicated by the small brace height increase, while attenuation slope might reflect a shift in inhibitory input patterns.

Complementarily, we investigated peak discharge rates and recruitment threshold because both are associated with motoneuron excitability and might also be influenced by PIC activity (Mesquita et al., 2024; Orssatto et al., 2022). Moderate and small decreases in peak discharge rates (*d *= −0.52) and recruitment threshold (*d *= −0.38) were observed in Control, contrasting with previous studies reporting no changes in tibialis anterior (Orssatto et al., 2022) or soleus (Mackay Phillips et al., 2023). Unlike those studies, participants in our study performed three randomised handgrip tasks with 5‐min intervals, possibly reducing carryover effects on Δ*F* but not on peak discharge rates or recruitment threshold. Furthermore, a small increase in peak discharge rates (*d *= 0.35) occurred only in 80%15s, while recruitment threshold increased in 40%15s (*d *= 0.42) and 40%30s (*d *= 0.26). Previous studies reported small peak discharge rates increases under 40%30s (Orssatto et al., 2022: *d *= 0.36; Mackay Phillips et al., 2023: *d *= 0.37), but while Orssatto et al. (2022) observed no changes in recruitment threshold, Mackay et al. (2023) did not report recruitment threshold results. Decreases in derecruitment thresholds were observed only in 40%15s (*d *= −0.61) and 80%15s (*d *= −0.33). The cause for non‐alignment with Δ*F* is unclear; however, since both recruitment and derecruitment thresholds rely on highly variable torque data, these variables should be interpreted cautiously. These findings suggest that PICs differentially modulate Δ*F*, peak discharge rates, and recruitment and derecruitment thresholds, each providing distinct insights into motoneuron excitability.

### 4.1 Final considerations and future directions

This study combined robust non‐invasive methods to estimate motor task influence on PIC activity in humans, likely via increased 5HT input to the spinal cord. Using an innovative EMG protocol we non‐invasively assessed key metrics of motoneuron excitability (e.g., Δ*F*, brace height and attenuation slope) without pharmacological intervention (Beauchamp et al., 2023). We also minimised the potential for type I error through rigorous statistical analysis (Yu et al., 2022). On the other hand, we assessed tibialis anterior MUs recruited during a ramp‐shaped contraction to 20% of maximal force, consistent with previous studies investigating the influence of remote contraction on ∆*F* (Mackay Phillips et al., 2023; Orssatto et al., 2022). This intensity is known to respond well to remote handgrip contractions and yield a higher number of motor units compared to higher contraction intensities (Orssatto et al., 2021). Thus, caution should be taken when extrapolating our findings to higher‐threshold units, at higher contraction intensities, and from different muscle groups. Additionally, direct in vivo 5HT measurements remain technically impossible, so indirect methods are necessary. Lastly, while this study focused on 5HT and NE contributions to PICs, other neuromodulators should be considered in future studies, e.g., spinal interneurons modulating motoneuron excitability via muscarinic m2 receptors, affecting resting K^+^ conductance (Miles et al., 2007).

Future studies could combine EMG with additional methods to clarify findings. For instance: (i) since both 40%30s and 80%15s conditions increased Δ*F* in young adults, similar protocols could assess neuromodulatory capacity in populations with potentially reduced 5HT input or impaired PIC activation, such as older adults, individuals with neurodegenerative diseases or partial spinal cord injuries; (ii) further investigations are desirable to find whether impulse‐specific effects on PIC modulation differ across muscle groups, as variations in motoneuron distribution may affect responsiveness to remote contraction‐induced neuromodulation; and (iii) although remote handgrip contractions significantly increased ∆*F* of tibialis anterior MUs, the functional significance for motor control and force production remains unclear. Recent work has suggested within‐subject associations between changes in ∆*F* and physical function following resistance training in older adults (Orssatto et al., 2023) and after short‐term unloading and active recovery in young adults (Martino et al., 2024). Also, between‐subject correlation of ∆*F* and physical function levels were observed in older adults (Orssatto et al., 2025). Even though there is evidence of a potential functional significance of ∆*F* levels on physical function, it is unclear whether the acute ∆*F* facilitation elicited by our experiment is sufficient to change motor output. Thus, further investigation into the functional role of acute ∆*F* modulation is warranted.

### 4.2 Conclusion

This study provides novel insights into the mechanisms through which remote voluntary muscle contractions influence motoneuron excitability, estimated by Δ*F* in tibialis anterior motor units. By manipulating both the intensity and the duration of a handgrip task, we identified contraction impulse as a critical factor driving changes in motoneuron recruitment–derecruitment hysteresis, which likely reflects increased PIC activity. Specifically, higher Δ*F* values in remote task conditions with matched contraction impulse suggest that both higher and lower intensity/duration conditions can lead to increased PIC modulation, provided that a sufficient impulse threshold is met. The increased brace height observed in the impulse‐matched conditions support our hypothesis that increased tibialis anterior Δ*F* following remote handgrip contraction may result from increased neuromodulation, possibly by augmented serotonergic input onto the spinal cord.

## AUTHOR CONTRIBUTIONS

The experiments for this study were conducted at the Faculty of Health, School of Exercise & Nutrition Sciences, Queensland University of Technology, Brisbane, Australia. The conception of the study was carried out by Lucas B. R. Orssatto and Gabriel S. Trajano The study design and data acquisition were performed collaboratively by Lucas Ugliara, Lucas B. R. Orssatto, and Gabriel S. Trajano Data analysis and interpretation were conducted by Lucas Ugliara, Lucas B. R. Orssatto, Amilton Vieira and Gabriel S. Trajano The manuscript was drafted by Lucas Ugliara and critically revised by Lucas B. R. Orssatto, Amilton Vieira and Gabriel S. Trajano All authors have read and approved the final version of this manuscript and agree to be accountable for all aspects of the work in ensuring that questions related to the accuracy or integrity of any part of the work are appropriately investigated and resolved. All persons designated as authors qualify for authorship, and all those who qualify for authorship are listed.

## CONFLICT OF INTEREST

None declared.

## Supporting information



Consistency of the participants in performing the triangular‐shaped contractions.

Sensitivity analysis including only the participants who successfully concluded all four conditions.

## Data Availability

The complete dataset and corresponding R script are accessible at https://github.com/lugliara/PICs.
