# Supplementary material for: Isometric handgrip contraction increases tibialis anterior intrinsic motoneuron excitability in a dose‐dependent manner
Source: Exp Physiol. 2026 Feb 3;111(4):2200–13. doi: 10.1113/EP092961 (PMC13140553; doi:10.1113/EP092961)
Supplement: Supplementary file 1 — Consistency of the participants in performing the triangular‐shaped contractions. [file EPH-111-2200-s002.docx]

**Supplementary Material 1.**

Consistency of the participants in performing the triangular-shaped contractions. We compared the area formed between the performed and the requested torque trace paths (Figure attached).

*Torque trace deviation from the requested path during the triangular contractions*

No Time by Condition interaction effect was observed [β = -0.66 (-17.0, 3.69) % of peak torque·s, SE = 5.28; t = -1.26]. The estimated marginal means (95%CI) for the area formed before and after intervention were: Control [63.8 (56.6, 71.0) % of peak torque·s; 59.1 (51.9, 66.2) % of peak torque·s], 40%15s [65.7 (57.9, 73.5) % of peak torque·s; 58.9 (51.1, 66.7) % of peak torque·s], 40%30s [61.8 (54.3, 69.3) % of peak torque·s; 63.6 (56.1, 71.1) % of peak torque·s], and 80%15s [64.0 (56.9, 71.2) % of peak torque·s; 66.0 (58.8, 73.1) % of peak torque·s].

We also compared the area formed between the performed and the requested torque trace paths during the ascending and descending phases. Regarding the area formed during the ascending and descending phases, no Time by Condition by Phase interaction effect was observed [β = 7.92 (-4.09, 19.9) %MVC, SE = 6.13; t = 1.29]. The estimated marginal means (95%CI) for the area formed, respectively before and after intervention, and for ascending and descending phases, were: Control [28.5 (23.9, 33.0) % of peak torque·s; 27.6 (23.0, 32.1) % of peak torque·s; 34.8 (30.3, 39.4) % of peak torque·s; 30.3 (25.8, 34.9) % of peak torque·s], 4015 [29.2 (24.2, 34.3) % of peak torque·s; 25.4 (20.3, 30.5) % of peak torque·s; 33.1 (28.0, 38.2) % of peak torque·s; 33.5 (28.5, 38.6) % of peak torque·s], 4030 [29.1 (24.3, 34.0) % of peak torque·s; 28.2 (23.4, 33.0) % of peak torque·s; 30.7 (25.8, 35.5) % of peak torque·s; 34.0 (29.1, 38.8) % of peak torque·s], 8015 [27.0 (22.5, 31.6) % of peak torque·s; 28.3 (23.8, 32.9) % of peak torque·s; 37.4 (32.9, 42.0) % of peak torque·s; 36.9 (32.4, 41.5) % of peak torque·s].

Additionally, we compared the area formed between requested and performed paths between included and excluded trials during the ascending and descending phases. The excluded trials were separated in trials excluded due to inconsistency during the ascending phase or during the descending phase.

Regarding trials excluded due to ascending phase, an Inclusion effect was observed [β = -8.12 (-15.0, -1.21) % of peak torque·s, SE = 3.526.13; t = -2.301.29]. The area was greater at excluded trials during the ascending phase [8.12 (1.21, 15.00) % of peak torque·s, d = 1.05 (0.16, 1.94)]. The estimated marginal means (95%CI) for the area formed were: included trials [28.0 (25.4, 30.7) % of peak torque·s]; excluded trials [36.2 (29.0, 43.3) % of peak torque·s].

Regarding trials excluded due to descending phase, an Inclusion effect was observed [β = -6.83 (-12.7, -0.99) % of peak torque·s, SE = 2.98; t = -2.29]. The area was greater at excluded trials during the descending phase [6.83 (0.99, 12.7) % of peak torque·s, d = 0.83 (0.12, 1.53)]. The estimated marginal means (95%CI) for the area formed were: included trials [34.0 (30.3, 37.6) % of peak torque·s]; excluded trials [40.8 (34.2, 47.4) % of peak torque·s].”

The data and scripts for this analysis have been made publicly available on <https://github.com/lugliara/PICs>.

*
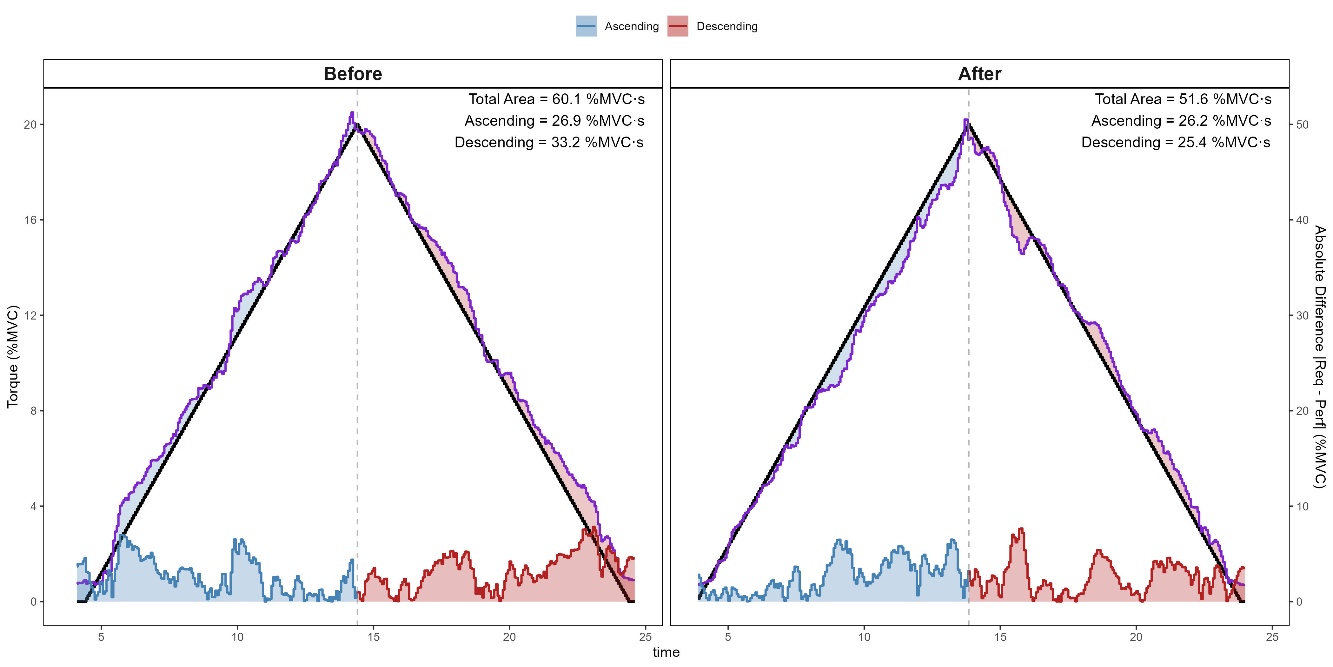
*
