# Supplementary material for: Isometric handgrip contraction increases tibialis anterior intrinsic motoneuron excitability in a dose‐dependent manner
Source: Exp Physiol. 2026 Feb 3;111(4):2200–13. doi: 10.1113/EP092961 (PMC13140553; doi:10.1113/EP092961)
Supplement: Supplementary file 2 — Sensitivity analysis including only the participants who successfully concluded all four conditions. [file EPH-111-2200-s001.docx]

**Supplementary Material 2.**

Sensitivity analysis including only the participants who successfully concluded all four conditions (N = 14).

*ΔF*

A time by condition interaction effect was observed β = −0.41 (−0.65, −0.17) pps, SE = 0.12; t = −3.39]. ΔF increased from before to after the intervention on 40%30s [0.30 (0.12, 0.49) pps, d = 0.44 (0.17, 0.71)] and 80%15s [0.34 (0.17, 0.51) pps, d = 0.49 (0.24, 0.74)], but remained unchanged on 40%15s [0.02 (−0.15, 0.19) pps, d = 0.03 (-0.22, 0.28)] and control [−0.08 (−0.24, 0.09) pps, d = -0.11 (-0.35, 0.13)] conditions (Figure 2 and 3).

*Brace height*

A time by condition interaction effect was not observed β = −2.27 (−5.15, 0.60) % rTri, SE = 1.47; t = −1.55].

*Attenuation slope*

A time by condition interaction effect was observed β = 0.06 (0.01, 0.10) pps/%MVT, SE = 0.02; t = 2.47]. Attenuation slope decreased from before to after the intervention on 40%30s [−0.05 (−0.09, −0.02) pps/%MVT, d = -0.29 (-0.49, -0.08)]and 80%15s [−0.03 (−0.07, −0.00) pps/%MVT, d = -0.34 (-0.53, -0.14)], but remained unchanged on 40%15s [−0.02 (−0.05, 0.02) pps/%MVT, d = -0.04 (-0.24, 0.15)] and control [0.01 (−0.03, 0.04) pps/%MVT, d = 0.10 (-0.08, 0.29)]s.

*Peak discharge rates*

A time by condition interaction was observed β = -0.65 (-0.80, -0.49) pps, SE = 0.08; t = -8.12]. Peak discharge rates decreased from before to after the intervention only on control [−0.35 (−0.46, −0.24) pps, d = -0.60 (-0.78, -0.41)] and increased on 80%15s [0.30 (0.18, 0.41) pps, d = 0.50 (0.31, 0.69)] condition, but remained unchanged on 40%15s [-0.01 (−0.13, 0.10) pps, d = -0.02 (-0.21, 0.17)] and 40%30s [−0.11 (−0.23, 0.01) pps, d = -0.19 (-0.39, 0.01)].

*Recruitment thresholds*

A time by condition interaction effect was observed β = -0.62 (-0.95, -0.29) % of peak torque, SE = 0.17; t = -3.72]. Recruitment thresholds increased from before to after the intervention only on 40%15s [0.45 (0.21, 0.68) % of peak torque, d = 0.37 (0.17, 0.56)] and 40%30s [0.36 (0.12, 0.60) % of peak torque, d = 0.29 (0.09, 0.49)] conditions, but remained unchanged on control [−0.17 (−0.40, 0.06) % of peak torque, d = -0.14 (-0.32, 0.05)] and 80%15s [0.18 (−0.05, 0.42) % of peak torque, d = 0.15 (-0.04, 0.34)] conditions.

*Derecruitment thresholds*

A time by condition interaction effect was observed β = 0.50 (0.21, 0.80) % of peak torque, SE = 0.15; t = 3.36]. Recruitment thresholds decreased from before to after the intervention only on 40%15s [−0.58 (−0.79, −0.36) % of peak torque, d = −0.52 (−0.71, −0.33)], 40%30s [−0.23 (−0.45, −0.01) % of peak torque, d = −0.21 (−0.41, −0.01)] and 80%15s [−0.22 (−0.43, −0.00) % of peak torque, d = −0.20 (−0.39, −0.00)] conditions, but remained unchanged on control [−0.07 (−0.28, 0.13) % of peak torque, d = −0.06 (−0.25, 0.12)] condition.

The data and scripts for this analysis have been made publicly available on <https://github.com/lugliara/PICs>.
